# Antiviral Therapeutic Potential of Curcumin: An Update

**DOI:** 10.3390/molecules26226994

**Published:** 2021-11-19

**Authors:** Abdollah Ardebili, Mohammad Hassan Pouriayevali, Sahar Aleshikh, Marziyeh Zahani, Mehdi Ajorloo, Ahdieh Izanloo, Abolghasem Siyadatpanah, Hadi Razavi Nikoo, Polrat Wilairatana, Henrique Douglas Melo Coutinho

**Affiliations:** 1 Laboratory Sciences Research Center, Golestan University of Medical Sciences, Gorgan 4934174515, Iran; aardebili2014@gmail.com; 2 Department of Microbiology, Faculty of Medicine, Golestan University of Medical Sciences, Gorgan 4934174515, Iran; saharal91@yahoo.com; 3 Department of Arboviruses and Viral Hemorrhagic Fevers (National Ref Lab), Pasteur Institute of Iran, Tehran 1316943551, Iran; mhpouriayevali@yahoo.com; 4 Student Research Committee, Faculty of Paramedicine, Golestan University of Medical Sciences, Gorgan 4934174515, Iran; marziyehzahani7697@gmail.com; 5 Hepatitis Research Center, Lorestan University of Medical Sciences, Khorramabad 6813833946, Iran; kmehdiajorloo@gmail.com; 6 Department of Virology, Lorestan University of Medical Sciences, Khorramabad 6813833946, Iran; 7 Department of Biology, Faculty of Science, Golestan University, Gorgan 4934174515, Iran; ahdieh.izanloo76@gmail.com; 8 Ferdows School of Paramedical and Health, Birjand University of Medical Sciences, Birjand 9717853577, Iran; asiyadatpanah@yahoo.com; 9 Infectious Diseases Research Center, Golestan University of Medical Sciences, Gorgan 4934174515, Iran; 10 Department of Clinical Tropical Medicine, Faculty of Tropical Medicine, Mahidol University, Bangkok 10400, Thailand; 11 Laboratory of Microbiology and Molecular Biology (LMBM), Regional University of Cariri (URCA), Crato 63105-000, CE, Brazil

**Keywords:** antiviral agent, curcumin, herbs, human viruses X

## Abstract

The treatment of viral disease has become a medical challenge because of the increasing incidence and prevalence of human viral pathogens, as well as the lack of viable treatment alternatives, including plant-derived strategies. This review attempts to investigate the trends of research on in vitro antiviral effects of curcumin against different classes of human viral pathogens worldwide. Various electronic databases, including PubMed, Scopus, Web of Science, and Google Scholar were searched for published English articles evaluating the anti-viral activity of curcumin. Data were then extracted and analyzed. The forty-three studies (published from 1993 to 2020) that were identified contain data for 24 different viruses. The 50% cytotoxic concentration (CC50), 50% effective/inhibitory concentration (EC50/IC50), and stimulation index (SI) parameters showed that curcumin had antiviral activity against viruses causing diseases in humans. Data presented in this review highlight the potential antiviral applications of curcumin and open new avenues for further experiments on the clinical applications of curcumin and its derivatives.

## 1. Introduction

According to the World Health Organization (WHO), infectious disease agents, such as bacteria, viruses, fungi, and parasites are estimated to be responsible for over 17 million deaths each year worldwide. Viruses are estimated to cause up to 390 million infections each year, with approximately 40% of the world’s population at risk of infection [1]. They are a leading cause of life-threatening diseases, a feature that makes them one of the largest health challenges worldwide. There are about 90 commonly known viral diseases affecting humans, from mild illnesses, including cold sore, gastroenteritis, flu, and warts, to severe forms, such as acquired immunodeficiency syndrome (AIDS), smallpox, dengue, zika, and respiratory syncytial virus (RSV) [2,3]. A number of reasons account for the difficulties with therapy for viral diseases, including the lack of drug efficacy and safety, the technically complex design of antiviral compounds, expensive production process, and emergence of new mutated variants [4,5] Additionally, some antiviral compounds are not always well tolerated. Drug-resistant strains of viruses have been found to be the main cause of therapeutic failures. HIV, influenza virus, and HSV, as the major agents responsible for more than 10 million cases of diseases worldwide, are the best example of phenotypes resistant to various antiviral drugs [6,7,8,9]. These viruses present an obvious requirement for the development of novel methods to treat viral infections, and to explore the repurposing of already approved pharmaceuticals or the use of natural substances. Herbal medicine refers to the development or application of natural compounds as a rich source of phytochemicals with different pharmacologic and therapeutic effects [10]. Several studies have been conducted to explore the antiviral potential of natural compounds. Accordingly, antiviral effects have been shown for components of green tea, cinnamon, numerous herbs, etc. [11,12]. Previous studies have found that curcumin has antiviral activity against different human viral pathogens, including both RNA and DNA viruses [13,14].

In this review, we intend to systematically update the antiviral properties of curcumin in vivo and in vitro. In addition, we also cover mechanisms of action of curcumin. Finally, we also discuss therapeutic trends and future perspectives regarding the use of curcumin, as well as novel and versatile classes of antiviral agents. In this regard, various electronic databases, including PubMed (https://www.ncbi.nlm.nih.gov/pubmed, accessed on 7 November 2021), Scopus (http://www.scopus.com, accessed on 7 November 2021), Web of Science (https://www.wofknowledge.com, accessed on 7 November 2021), and Google Scholar (https://scholar.google.com, accessed on 7 November 2021) were searched. The following MeSH (Medical Subject Headings) terms and keywords were used: “curcumin AND antiviral AND in vitro”, “curcumin AND antiviral AND in vivo”, “curcumin AND viral infection AND in vitro activity”, “curcumin AND viral infection AND in vivo activity”, “curcumin AND phytochemical AND viral infection”, “curcumin AND enveloped viruses”, “curcumin AND non-enveloped viruses”, “curcumin AND DNA viruses”, “curcumin AND RNA viruses”, “curcumin AND herb AND antiviral”. Articles were retrieved through titles and abstracts. Data were extracted in terms of publication year, study geographic location, study scope, viruses tested, cytotoxicity tests, antiviral testing methods, and the mode of action of curcumin.

## 2. Curcumin

Curcumin (1,7-bis(4-hydroxy-3-methoxyphenyl)-1,6-heptadiene-3,5-dione), also called diferuloylmethane, is the best example of a plant derivative with an enormous number of therapeutic properties, such as anti-oxidant, anti-carcinogenic, anti-diabetic, anti-microbial, and antiviral activity [12]. In traditional Indian Ayurvedic medicine, curcumin was widely applied in many therapeutic remedies [13]. This compound is a natural polyphenolic substance and an active form of the traditional herb that is found in the rhizome of Curcuma longa (turmeric) and in other Curcuma spp, and that is commonly used as a spice and coloring agent in food [15]. Curcumin is the main molecule of the curcuminoids; the curcuminoids are comprised of curcumin (77%) as well as includes bisdemethoxycurcumin (BDMC) (17%) and demethoxycurcumin (DMC) (6%) [16]. For the purpose of this review, we will refer to the purified products by name, and curcuminoid will refer to two or more of these compounds together.

The first suggestion that curcumin had antiviral properties came in the 1990s, with the discovery that curcumin and curcumin boron complexes could inhibit the human immunodeficiency virus (HIV) viral protease in vitro, with an average inhibitory concentration (IC50) of 100 μM [17]. Since then, numerous studies have found that curcumin has antiviral activity against a diverse set of viruses, including both RNA and DNA viruses, both enveloped and non-enveloped, as is systematically updated in detail below [12,14].

## 3. Selected Studies

The main characteristics of the 46 selected studies are presented in Table 1. Articles were published from July 1993 to November 2021. Multiple selected studies were on different types of human viruses, including human immunodeficiency virus (nine studies), hepatitis C virus (five studies), human cytomegalovirus (three studies), hepatitis B virus (four studies), herpes simplex viruses (four studies), dengue virus (four studies), enterovirus 71 (two studies), human T lymphocyte virus (two studies), vesicular stomatitis virus (two studies), and respiratory syncytial virus (two studies). There was one study for viruses including zika and chikungunya, coronavirus, Rift Valley fever virus, human norovirus, coxsackievirus B3, Japanese encephalitis virus, and viral hemorrhagic septicemia virus. To determine the cytotoxicity effect of curcumin, MTT (3-(4,5 dimethyl thiazoleyl -2)- 2,5-diphenyl tetrazolium bromide) and trypan blue exclusion assays were used in eighteen and four studies, respectively; whereas the water-soluble tetrazolium salt (WST) and cell counting kit-8 (CCK8) method was used in two studies. These assays are dependent on the number of viable cells and the value is referred to as the median cellular cytotoxicity concentration (CC50). In all included studies, different dilutions of curcumin were evaluated to determine cytotoxicity concentration. The number of viable cells was directly determined by colorimetric methods and the 50% cytotoxicity concentration (CC50) was calculated by nonlinear regression analysis. Cell culture methods used for the evaluation of the cytotoxicity concentration were performed according to cell culture guidelines. Accordingly, CC50 values were determined in 36 studies. Others selected various concentrations of curcumin based on the observed low toxicity and cell viability decrease in a dose-dependent manner. Twelve, seven, thirteen, and one studies used plaque reduction assay, TCID50, MTT, hemagglutination inhibition assay (HI), and Immunofluorescence (IFA) methods, respectively, for in vitro antiviral activity of curcumin. The value of the minimum concentration of curcumin is referred to as the median effective or inhibitory concentration (EC50/IC50) to reduce a 50% cytopathic effect (CPE) and was calculated by linear regression analysis. Therapeutic index (TI) or selectivity index (SI) was expressed as the ratio of CC50/EC50. Virological methods used for the evaluation of viral titration were performed according to standard guidelines. Of the 43 studies reviewed, sixteen studies reported data on the EC50 or IC50 values of curcumin to the different viruses. Furthermore, 27 remaining studies showed that curcumin reduced the production of infectious particles in various infected cells in a dose-dependent manner.

## 4. Antiviral Activity of Curcumin against RNA Viruses

**Figure 1:**
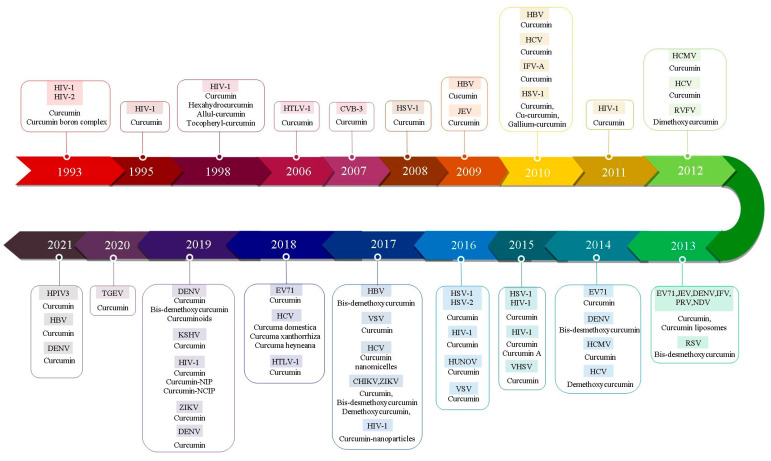
Timeline of development of curcumin therapy against different types of human viruses: IAV, influenza A virus; PIV-3, parainfluenza virus 3; ZIKA, Zika; CHIKV, Chikungunya virus; JEV, Japanese encephalitis virus; EV71, enterovirus 71; HCV, hepatitis C virus; VSV, vesicular stomatitis virus; EV, Ebola virus; RSV, respiratory syncytial virus; HIV, human immunodeficiency virus; HTLV-1, human T-lymphocyte virus; RVFV, Rift Valley fever virus; HuNoV, human norovirus; CVB3, coxsackievirus B3; TGEV, transmissible gastroenteritis virus; HBV, hepatitis B virus; HSV, herpes simplex viruses; HCMV, human cytomegalovirus; EBV, Epstein–Barr virus.

Data from recent research into the antiviral properties of curcumin toward various RNA viruses, including influenza A virus (IAV), parainfluenza virus 3 (PIV-3), Zika (ZIKA), Chikungunya virus (CHIKV), Japanese encephalitis virus (JEV), enterovirus 71 (EV71), hepatitis C virus (HCV), vesicular stomatitis virus (VSV), Ebola virus (EV), respiratory syncytial virus (RSV), human immunodeficiency virus (HIV), human T-lymphocyte virus (HTLV-1), Rift Valley fever virus (RVFV), human norovirus (HuNoV), and coxsackievirus B3 (CVB3) were collected (Table 1, Figure 1).

### 4.1. HCV

Curcumin components with αβ-unsaturated ketone groups reduce membrane fluidity of HCV, leading to inhibition of virus attachment and fusion to cells. Accordingly, curcumin inhibits the entrance of all HCV genotypes to cells tested in a dose-dependent manner with a half-maximal inhibitory concentration (IC50) of about 8.46 ± 1.27 μM [17,18,39,61]. Other studies also demonstrated that curcumin can inhibit RNA replication and NS5A and NS5B expression of HCV in infected cell lines via suppression of the PI3K-AKT and Akt-SREBP-1 pathways and induction of heme oxygenase [29,32,46].

### 4.2. Zika Virus

Curcumin can be suppressive when added to cells before and after Zika or chikungunya infection, although curcumin acts against Zika exclusively during cell-attachment or entry and not at later stages of infection. Mounce et al. showed that 5 μM curcumin was more effective when added before infection and decreased the viral titer by more than 0.5 log10 without any cytotoxicity effects. Curcumin had also an IC50 of 1.9 μM and 3.89 μM for Zika and chikungunya, respectively [48]. Furthermore, they found that curcumin prevented the entry or attachment of chikungunya virus (CHIKV) to host cells, but that it has no impact on the viral replication machinery [48,62]. In general, data reported from this study indicates that curcumin likely inhibits these viruses directly through its effect on viral surface glycoproteins and by altering the conformation of viral surface proteins [48,62].

### 4.3. Dengue Virus

Regarding the activities of curcumin against arboviruses, it has been shown that curcumin inhibits dengue virus propagation in a dose-dependent manner that might be due to an increase of Lys48 ubiquitin-conjugated proteins and accumulation of viral proteins. The anti-dengue effect of curcumin was also evaluated on BHK-21 cells infected with dengue 2 virus. The CC50 and IC50 of treated BHK-21 cells with curcumin were 29.5 μM and 11.51 μM, respectively [62]. The anti-dengue activity of curcumin has been evaluated by four studies [34,53,56,59]. A recent study in this review evaluated the inhibitory effect of the same selection of compounds against dengue virus (DENV) [62]. Gao et al. also found that curcumin significantly reduced plaque formation of all four strains (DENV-1-4, IC50 of 9.37, 3.07, 2.09, and 4.83 μM, respectively), with limited cytotoxicity effects (CC50 of 59.42 μM). Though the mechanism of action was not addressed [48], another study demonstrated that curcumin likely inhibits DENV-2 indirectly through its impact on cellular systems, rather than directly on the virus [56]. In an in vitro study conducted by Balasubramanian et al., curcumin, bisdemethoxycurcumin, and three other synthesized analogues potentially inhibited viral protease activity (IC50 of ~36–66 μM). Their compounds only modestly inhibited replication of a DENV2 reporter replicon construct, with the acyclic and cyclohexanone analogues of curcumin performing slightly better than the natural curcuminoids (50% effective concentration (EC50) of 8.61 and 8.07 μM versus 13.91 μM) [53]. They demonstrated that curcumin and other synthesized analogues likely inhibit DENV-2 indirectly through their impact on cellular lipid metabolism, such as acetyl-CoA carboxylase, fatty acid synthase, and lowered lipid droplet (LD) formation [53].

### 4.4. JEV

Curcumin at a concentration of 5 μM significantly increased viability in JEV-infected cells, so that the results of the terminal deoxynucleotide transferase-mediated dUTP nick-end labeling (TUNEL) assay showed that the apoptotic pattern of JEV-infected cells treated with curcumin reduced compared to the control group. Pre-treatment and co-treatment of infected cells with curcumin (10 μM) inhibited JEV plaque formation, while no change was observed when curcumin was added after 2 hours of infection, indicating the blocking function of curcumin on envelope proteins. The inhibitory effects of curcumin was found to be its suppression of the proteasome system, downregulating the reactive oxygen level, modulating the membrane integrity and cellular stress proteins level, and inhibiting pro-apoptotic signaling molecules [25,34].

### 4.5. RSV

Curcumin at concentrations ranging from 5 to 15 μM has been found to reduce the expression of the RSV N protein by 50 to 90%, respectively. Without any direct effect on the expression of cellular receptors and RSV binding process, curcumin inhibited viral infection during the entry and fusion phase [63]. Obeta et al. showed that both replication and expression of structural proteins in RSV were suppressed with 10 μg/mL of curcumin by increasing the protein kinase R expression and the phosphorylation of NF-kB and eIF-2a. Curcumin also prevented the epithelial inflammatory responses in human nasal epithelial cells by downregulation of cyclooxygenase-2 (COX2) [35].

### 4.6. EV71

Two studies in this review evaluated the inhibitory effect of curcumin on enterovirus 71 [17,51].It has been found that enterovirus 71 showed significant abrogated viral proteins and reduced viral titer by about 6 log10 (10^6^ fold) in the presence of curcumin at a concentration of 40 μM at early infection. One study revealed that curcumin reduced the activity of enterovirus-induced ubiquitin-proteasome without any effect on antioxidant activity and the interference of ERK. In addition, curcumin downregulates GBF1 and PI4KB, both of which are required for the formation of the viral replication complex. Anti-apoptotic properties of curcumin are related to decreases of PARP-1 and cleaved caspase-3 [17]. In the second study, curcumin induced PKCδ phosphorylation in intestinal epithelial cells, a process which is important for the replication of EV71 and protein expression [51].

### 4.7. IFVA

There are two studies reporting data on the antiviral activity of curcumin against the influenza A virus. Curcumin at a 30 μM concentration showed a 90% decrease in influenza viral load in the infected Madin–Darby canine kidney (MDCK) cell line, while the EC50 and CC50 in MDCK were 0.47 μM and 43 μM, respectively. A timely assessment of drug-addition revealed a direct effect of curcumin on H1N1 and H6N1 infectivity through blocking of hemagglutination [27]. Another study revealed a significant decrease in the infectivity rate of enveloped viruses such as the influenza virus, Japanese encephalitis virus, and dengue virus with 30 μM of curcumin (EC50: 0.47 μM), which was not effective on non-enveloped viruses such as enterovirus. Taken together, these studies demonstrate curcumin’s potential against enveloped viruses [34]. IAV needs NF-KB signaling to replicate, and curcumin inhibits this signaling [64]. Curcumin interrupts virus–cell attachment, which leads to the inhibition of influenza virus propagation [64]. Curcumin and its analogues can inhibit IAV by preventing entry and exit of viruses, and oral therapy with curcumin improved the survival of IAV-infected mice [65].

### 4.8. HIV

Three studies in this review evaluated the antiviral activity of curcumin on HIV. In one study, curcumin degraded the Tat protein via the proteasome pathway and reduced Tat-dependent transactivation and replication in HIV-1 infected cells [20]. Curcumin significantly prevented the disruption of tight junction proteins and protected the epithelial barrier. On the other hand, pretreatment and co-treatment with curcumin significantly inhibited the induction of proinflammatory cytokines (Il-6, TNF) or chemokines (IL-8, IP-10, RANTES, MCP-1, MIP-1α, and eotaxin) [50]. Another study showed that curcumin can reduce inflammation in the female genital area, which allows easier infection by HIV [40].

### 4.9. Coxsackievirus

One study assessing the antiviral effect of curcumin showed that it reduces the expression and replication of coxsackievirus in infected HeLa cells. This study demonstrated that such antiviral effects were achieved by dysregulation of the ubiquitin–proteasome system (UPS) and inhibition of UPS activity by about 30% [22,66].

### 4.10. VSV

One study assessing the antiviral effect of curcumin showed that it reduces the replication of VSV in infected Vero cells. This study demonstrated that such antiviral effects were achieved by over-expression of Dicer-1 in VSV-EGFP infected cells, in comparison with the control (DMSO) [47]. They found that 10 μM of curcumin provided robust inhibition of recombinant VSV-EGFP infection of Vero cells, as measured via plaque assay and fluorescence, with approximately 33% reduced infection at MOI 0.0002 and a nearly 90% reduction at MOI 0.00002 after 24 h [45,67].

### 4.11. Coronavirus

Curcumin can inhibit SARS-CoV replication with EC50 >10 μM (40). Furthermore, several studies suggest that curcumin can inhibit SARS-CoV-2 replication [68,69]. Curcumin can block the interaction between the spike glycoprotein and angiotensin-converting enzyme 2 (ACE2) and inhibit the Nsp15 protein, therefore blocking replication of the virus or inhibiting viral protease [70,71,72]. These observations were supported by a study by Han et al. who demonstrated that curcumin strongly inhibited TGEV proliferation and viral protein expression in a dose and time-dependent manner, and treatment with curcumin caused a reduction in both viral particles (IC50 of 8.6 μM) and protein levels in porcine kidney cells. This study suggested that curcumin may inhibit the adsorption of TGEV or that it possesses excellent virucidal activity [57].

### 4.12. Norovirus

For enveloped viruses, direct incubation with curcumin frequently disrupts the membrane integrity and ability of the virus to bind to cells by blocking the action of surface glycoproteins on the virus [14,34]. One study showed that curcumin reduces the infectivity of human norovirus by 91% in human norovirus (HuNoV) replicon-bearing HG23 cells. This study suggested that curcumin may involve viral entry or affects virus particle integrity and does not alter other aspects of the virus lifecycle [73].

### 4.13. Human Parainfluenza Virus Type 3

The anti-HPIV3 activity of curcumin was evaluated by one study. This study showed that curcumin disrupts F-actin, resulting in reduced viral inclusion body (IB) formation and inhibiting virus replication [58].

## 5. Antiviral Activity of Curcumin against DNA Viruses

Data from the recent research about the antiviral properties of curcumin toward various DNA viruses, including the hepatitis B virus (HBV), herpes simplex viruses (HSV-1 and 2), human cytomegalovirus (HCMV), Epstein-Barr virus (EBV), and human papillomavirus (HPV) were collected.

### 5.1. HBV

The anti-HBV activity of curcumin was evaluated by several studies. Aqueous extract of Curcuma longa Linn (CLL) in 200 mg/L and 500 mg/L caused a reduction of about 80% in HBsAg and HBV particle production compared with non-treated controls. This effect might be due to the specific inhibitory effect of CLL in HBV replication followed by repression of RNA transcription. Interestingly, CLL extract in a dose-dependent manner inhibited HBV enhancer I and X transcription by more than 80% through increasing expression and prolonged stability of p53 [24]. Wei et al. showed that 20 μmol/L of curcumin effects led to about 57% and 75.5% repression of HBs Ag and HBV cccDNA levels, respectively, without any cytotoxicity, compared with the control, which might be via reduction of cccDNA-bound histone acetylation [49].

Further studies showed that the phenolic compound of curcumin suppresses HBV replication via reduction and degradation of the PGC-1a protein, a key factor of gluconeogenesis, which induces HBV expression. Furthermore, the combination of curcumin and anti-HBV reverse-transcriptase lamivudine reduces HBV expression by approximately 75% [26]. Additionally, one study suggested that curcumin may interrupt viral entry and suppress HBV re-infection [59].

### 5.2. Adenovirus

In two studies, the effect of curcumin on human adenoviruses was investigated. The A549 human lung adenocarcinoma cells were infected with human adenovirus types 4, 5, and 7 and the effect of curcumin showed that curcumin reduced the expression of viral early protein 1 (E1A) in several types of this virus. Curcumin also reduced the genome copy number of virus that were determined with plaque assay [74,75].

### 5.3. HSV

Curcumin inhibited HSV immediate early (IE) gene expression and infection. Curcumin, without interfering in HSV genome entry to the nucleus and VP16 binding to IE gene promoters, leads to reduced linkage of RNA polymerase II to promoters, although this effect was observed in the low concentration required to inhibit global H3 acetylation [23]. There are two studies reporting data on the antiviral activity of curcumin against herpes viruses. In a study by Flores et al., different concentrations of curcumin were investigated and the results showed that the minimum inhibitory concentration was 30 μM in the HSV1&2 infected Vero cell line. At this concentration, curcumin blocked viral adsorption and inhibited plaque formation about 92% and 88%, respectively. Curcumin and its derivatives, such as gallium-curcumin and Cu-curcumin showed also similar antiviral effects in vero cell line [43]. Anti HSV-2 activity of curcumin was evaluated in primary human GECs. Curcumin at a 5 μM concentration reduced viral replication 1000-fold in comparison to the control group and 50 μM of curcumin had a inhibitory property of 100% [76].

### 5.4. HCMV

An in vivo study of HCMV showed that curcumin can reduce anti-CMV antibody levels and viral load, and inhibit CMV pathological changes of the liver, kidneys, and lungs in an infected animal model. High (25 μg/mL) and middling (10 μg/mL) doses of curcumin can significantly inhibit CMV-induced apoptosis in an in vitro study [31].

## 6. Discussion

**Figure 2:**
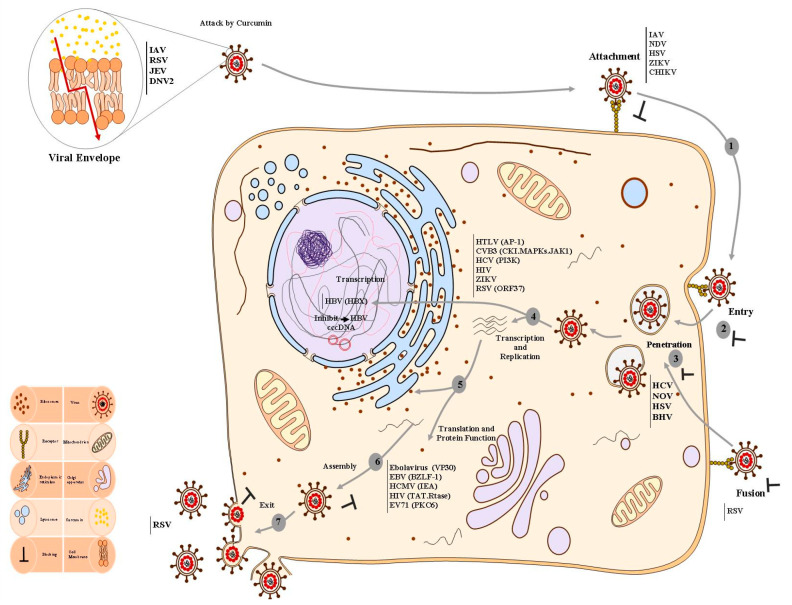
The effects of curcumin on different viruses and multi-site inhibitory effects of curcumin in the life cycle of human viruses. In general, the virus life cycle can be divided into various stages including: (**1**) attachment of virion, (**2**) entry, (**3**) viral genome replication, (**4**) viral transcription, (**5**) viral translation, and (**6**) virion assembly and exit. Hence, these critical steps specific to the viral life cycle have been attractive targets for chemotherapeutic intervention. Pathways and processes are inhibited by curcumin and its analogues, which affect various stages of the virus life cycle. Curcumin blocks viral attachment and entry in several enveloped viruses by abrogating the function of viral envelope proteins. Furthermore, curcumin serves as a veridical agent via attacking and disrupting the integrity of viral membrane envelopes. Additionally, curcumin influences viral replication machinery in two ways: (**i**) directly targeting the viral replication machinery, and (**ii**) interrupting viral replication machinery through modulating host cell signaling pathways, for instance, NF-κB, PI3K-AKT, Jab-1, and inflammation, as well as transcription/translation factors, which then cardinally hinder virus replication. The versatile anti-viral effect of curcumin has been demonstrated in numerous viruses as indicated in the boxes. IAV, influenza A virus; PIV-3, parainfluenza virus 3; CHIKV, chikungunya virus; JEV, Japanese encephalitis virus; EV71, enterovirus 71; HCV, hepatitis C virus; VSV, vesicular stomatitis virus; EV, Ebola virus; RSV, respiratory Syncytial virus; HIV, human immunodeficiency virus; HTLV-1, human T-lymphocyte virus; RVFV, Rift Valley fever virus; HuNoV, human norovirus; CVB3, coxsackievirus B3; HBV, hepatitis B virus; HSV, herpes simplex viruses; HCMV, human cytomegalovirus; EBV, Epstein–Barr virus.

Given the increasing global incidence of viral infections, as well as the lack of preventive and therapeutic options, there is an urgent need for new anti-viral drug approaches to be elucidated. Curcumin is known today as a “highly effective natural compound” against several viruses [3]. Here, we investigated published studies about the in vitro antiviral activity of curcumin to better understand its properties on different types of viruses. This can help the scientific community to design effective infection control programs for the eradication of viral infections. According to studies in this review, curcumin showed potent activity against a wide range of viruses tested, such that all studies found that their included viruses were susceptible to this compound. There are several steps in the virus replicative cycle, including attachment/penetration, uncoating, genome replication, gene expression, assembly, and release, and each process may serve as an attractive target for chemotherapeutic intervention. In the present review, we showed different mechanisms-of-action (MOA) of curcumin and also discussed the target indications, as shown in Figure 2.

In the attachment step, infectious particles enter host cells by attaching to receptors on the host cell membrane surface to promote uptake by receptor-mediated endocytosis [27,34]. Reductions of infectious viral loads in several enveloped viruses treated with curcumin were found in numerous studies, indicating the inhibitory effect of curcumin on viral envelope proteins [34]. As was first described by Li et al., curcumin affects the membrane lipid bilayer as a modulating agent [77]. Additionally, numerous studies have shown that curcumin inhibits the entry of the different viruses into cell and particle production by its interaction with the viral surface proteins [39,41,46]. Regarding the effect of curcumin on virus entry, eight studies in the present review reported that curcumin can potentially inhibit the uptake of viruses and reduce viral particle production [34,38,73]. Chen et al., found a 90% decrease in influenza viral load in infected MDCK cell lines treated with 30 μM curcumin. In addition, they showed a direct effect of curcumin on H1N1 and H6N1 infectivity through the blocking of hemagglutination [27]. A recent study indicated that curcumin inhibits enveloped virus infectivity, such as the influenza A virus, dengue virus type II, and Japanese encephalitis virus (JEV), through disruption of the integrity of viral membranes [34]. Particularly, this study showed that the EC50 value of curcumin in terms of inhibition of plaque formation for larger viruses is greater than that for smaller viruses (1.15 μM and 4.61 μM for influenza and PRV, respectively) [34]. Another study revealed that curcumin blocks the entry of CHIKV (Tongaviridae) and Zika virus by inhibiting the binding of viruses to host cells. In particular, they found a significant decrease in viral titers in a dose-dependent manner, so that concentrations at or above 100 nM showed effective antiviral activity in infected cells compared to untreated controls [48]. With the exception of two studies, which reported a 91% decreased viral load of human norovirus (HuNoV) as a non-enveloped virus, others confirmed that curcumin blocks the entry of viruses, or disrupts the integrity of the membranes of viral envelopes [48,78].

It has been shown that curcumin influences viral replication machinery in two ways: (i) direct targeting the viral replication machinery, and (ii) interruption of viral replication machinery through modulating cellular factors [24,33,71,78]. In 5 studies included in this review, the inhibitory effects of curcumin on HIV-integrase, protease as well as trans-activator factor Tat was evaluated [17,18,19,30,50]. Two studies revealed that curcumin interacts with the active sites of HIV protease and integrase. One of the five studies reported that curcumin treatment inhibited 55% of Tat-dependent transcription of HIV [18,66]. In addition to the direct targeting of viral proteins, curcumin can reduce the production of the HIV-1 virion in transfected HEK-293T cell line that treated with 80 μM curcumin from 0–8 hrs. In general, data reported from their study indicate that the viral p24 level in infected TZM-bl cells also decreased by 30% at a curcumin concentration of 20 μM and reached up to 90% at an 80 μM concentration [18,19].

Several distinct modulating cellular pathways have been described as responsible for antiviral effects mediated by curcumin. Three studies showed antiviral effects of curcumin against hepatitis viruses [26,28,29,32]. One study reported that the proteins level of HBV, such as HBsAg and core, were decreased by 73% and 45%, respectively, in stable transfected hepatoma cells [26]. They found that treatment of HBV with curcumin significantly suppressed HBV gene expression and replication through the downregulation of a coactivator of key gluconeogenesis pathway, PGC1α, resulting in suppressed HBV transcription [26]. In two different studies, curcumin reduced the replication of HCV by suppressing cellular factors, such as AKT-SREBP-1, ERK, and NF-κB [29,32]. One study reported that curcumin at a 25 μM concentration inhibited replication by suppressing the AKT pathway, which, in turn, suppressed the transcriptional factors, such as ERK and NF-κB. The second study showed that curcumin decreases HCV gene expression by suppression of AKT-SREBP-1, not by NF-κB [29]. Another study revealed that curcumin inhibits the replication of Rift Valley fever virus (RVFV; Phenuiviridae) by interfering with IKK-2-mediated phosphorylation of the viral protein NSs, as well as by altering the cell cycle of the treated cells. Notably, this did not only hold true in vitro, but also in mice subcutaneously treated with curcumin, which showed increased survival (60% compared to untreated animals) and decreased hepatic viral load (90% compared to controls) [33]. A recent study has shown that curcumin inhibits the replication of recombinant VSV-EGFP by increasing the expression level of Dicer-1. They found that treatment of recombinant VSV-EGFP with 10 μM of curcumin significantly reduced infection at MOI 0.0002, with a nearly 90% reduction at MOI 0.00002 after 24 h [47].

Dysregulation of UPS appears to be one of the general mechanisms by which curcumin restricts infection of viruses, including two flaviviruses, Japanese encephalitis virus (JEV), dengue virus type 2 (DNV-2), two enteroviruses, EV71, and coxsackievirus B3 (CVB3) [22,25,51,53].

## 7. Conclusions

Based on evidence obtained from this review, curcumin is known today as a “highly effective natural compound” that also has adequate in vitro activity against a wide range of viruses, as tested through various mechanisms. Although curcumin showed potent activity with no or minimal toxicity, it has low bioavailability and is rapidly metabolized. To overcome these drawbacks, several nanoparticle compounds with enhanced efficacy has been developed. Curcumin with enhanced efficacy has been developed through nanoparticle-based approaches (liposomes or micelles) and matrix-based formulations (hydrogels and nano-emulsions), resulting in increased absorption and/or bioavailability of curcumin than with unenhanced curcumin. Although extensive studies have been performed in a detailed manner to identify different molecular and cellular mechanisms of curcumin against viruses, some of these mechanisms are unclear, hampering the use of curcumin in clinics. Since the clinical efficacy of curcumin remains a matter of controversy, and only a few clinical trials have evaluated the safety, pharmacokinetics, and antiviral effectiveness of curcumin, more primary research articles and clinical trials are necessary.

## Figures and Tables

**Table 1:** Characteristics of selected studies on the anti-viral activity of curcumin and its derivatives.

Year	Virus	Study Scope	Substance	CC50 ^1^	IC50 ^2^	SI ^3^	Method	Mechanism of Action	Ref
1993	Human immunodeficiency virus 1 and 2 (HIV-1 and -2)	Evaluation of curcumin and curcumin boron complexes on the HIV-1 and HIV-2 proteases	Curcumin, Curcumin boron complex	1–500 μM	HIV1 (100 μM), HIV2 (250 μM)	ND ^4^	HPLC ^5^, SDS-PAGE ^6^, Fluorescent test	Inhibition of viral proteases	[18]
1995	Human immunodeficiency virus 1 (HIV-1)	Evaluation of curcumin on HIV-1 integrase	Curcumin	10–100 μM	40 μM	ND	PAGE	Inhibition of HIV-1 integrase	[19]
1998	Human immunodeficiency virus 1 (HIV-1)	Determination of curcumin and curcumin derivatives activity on HIV-1 Tat protein	Curcumin, Hexahydrocurcumin, Allyl-curcumin, Tocopheryl-curcumin	1 μM, 5 μM, 100 μM	ND	ND	Trypanblue, Oշ-assay	Reduction of Tat-mediated HIV transcription, leading to inhibition of replication	[20]
2006	Human T lymphotropic virus 1 (HTLV-1)	Determination of curcumin effect on AP-1 in HTLV-1	Curcumin	50 μM	ND	ND	Western blot	Inhibition of the constitutive AP-1 ^7^ activity and viral transcription by downregulation of JunD protein	[21]
2007	Coxackivirus B3 (CVB3)	Evaluation of curcumin on replication oxsackievirus B3	Curcumin	30 μM	ND	ND	Plaque assay, Western blot	Reduction of CVB3 replication by inhibition of intracellular signaling pathways, including MAPKs ^8^, CKII, and Jab1 ^9^	[22]
2008	Herpes simplex virus-1 (HSV-1)	Evaluation of curcumin on herpes simplex virus immediate-early gene expression	Curcumin	20 μM	25 μM	ND	Plaque assay, PCR ^10^ Western blot, Real-time PCR	Interference of VP-16 mediated recruitment of RNA polymerase II to immediate-early gene promoters	[23]
2009	Hepatitis B virus (HBV)	Determination of antiviral effect of *Curcuma longa* Linn extract on hepatitis B virus replication	Curcumin	200 μg/L, 500 μM	ND	ND	MTT ^11^, Southern blot, RT-PCR ^12^, Western blot	Enhancing the cellular accumulation of p53 protein, and repression of the HBx gene replication and transcription process	[24]
2009	Japanese encephalitis virus (JEV)	Determination of curcumin activity on Japanese encephalitis virus infectivity	Curcumin	5 μM, 10 μM	Dose-dependent	Dose-dependent	MTS ^13^, Plaque assay	Decreases ubiquitin proteasome system, causing the reduction of infective viral particle production	[25]
2010	Hepatitis B virus (HBV)	Evaluation of curcumin on hepatitis B virus replication	Curcumin	50–150 μM	Dose-dependent	Dose-dependent	Western blot	Suppression of HBV expression in a PGC-1a ^14^ dependent manner	[26]
2010	Influenza virus (IFV-A)	Evaluation of curcumin on influenza virus infection and hemagglutination	Curcumin	43 μM	0.47 μM	92.5 μM	Plaque assay, Western blot, HI ^15^	Interruption of virus attachment	[27]
2010	Herpes simplex virus-1 (HSV-1)	Evaluation of antiviral activities of curcumin derivatives against HSV-1	Curcumin, Gallium-curcumin, Cu-curcumin	484.2 μM, 255.8 μM, 326.6 μM	33.0 μM, 13.9 μM, 23.1 μM	14.6 μM, 18.4 μM, 14.1 μM	TCID50 ^16^ Trypanblue	Antiviral effects on HSV-1 in cell culture.	[28]
2010	Hepatitis C virus (HCV)	Evaluation of curcumin activity on the replication of hepatitis C virus	Curcumin	5–15 μM	Dose-dependent	Dose-dependent	MTT assay RT-PCR	Inhibition of HCV replication via the PI3K ^17^/Akt and SREBP-1-pathway, not NF-kB ^18^ pathway	[29]
2011	Human immunodeficiency virus 1 (HIV-1)	Determination of curcumin effect on HDAC1/NFκB in HTLV-1	Curcumin	ND	ND	ND	MTT	Inhibition of Tat-regulated transcription, by targeting cellular factors such as AMPK/HDAC1/NFκB.	[30]
2012	Human cytomegalovirus (HCMV or HHV5)	Evaluation of in vitro activity of curcumin on HCMV	Curcumin	25 μM, 10 μM, 1 μM	10	ND	MTT, TCID50	Decreases viral DNA and apoptosis in the infected cells	[31]
2012	Hepatitis C virus (HCV)	Evaluation of curcumin on HCV replication	Curcumin	5–25 μM	Does–dependent	ND	MTT, Real-time PCR, Western blot	Inhibition of viral replication by induction of the HO-1 expression and the inhibition of the PI3K-AKT signaling pathway	[32]
2012	Rift Valley fever virus (RVFV)	Evaluation of curcumin on Rift Valley fever virus replication	Demethoxycurcumin	10 μM	Time-dependent	ND	Western blot, RT-PCR, Plaque assay	Inhibition of NF-κB transcription factor	[33]
2013	Enterovirus 71 (EV71), Japanese encephalitis virus (JEV), Dengue virus (DENV), Influenza virus (IFV), Pseudorabies viruses (PRV), Newcastle disease viruses (NDV)	Evaluation of curcumin on enveloped viruses’ infectivity	Curcumin, Curcumin liposomes	30 μM, 62.5 μM	4 μM	ND	Plaque assay, HI, MTT	Disruption of the integrity of the viral membrane envelopes and liposomes.	[34]
2013	Respiratory syncytial virus (RSV)	Evaluation of curcumin on replication of RSV	Bis-desmethoxycurcumin	5 μM	ND	ND	MTT, Real-time PCR, Western blot, RT-PCR, ELISA ^19^	Prevention of viral replication, budding process, and reduction of cell pro-inflammatory responses. Inhibition of NF-κB transcription factor and eIF-2a	[35]
2014	Enterovirus-71 (EV71)	Evaluation of curcumin on the replication of enterovirus 71	Curcumin	40 μM	ND (6 log decrease)	ND	TCID50, RT-PCR, Western blot	Inhibition of viral replication by downregulation of the GBF1 ^20^ and PI4KB ^21^ in EV71- infected cells. Curcumin suppressed UPS ^22^ and apoptosis in EV71-infected cells.	[17]
2014	Human cytomegalovirus (HCMV or HHV5)	Evaluation of curcumin on the cytomegalovirus replication	Curcumin	0/2–0/8 μg	ND	ND	ELISA, Flow cytometry, Real-time PCR, IF ^23^, Western blot	Downregulation of the gene expression of HCMV immediate early and UL83 genes by curcumin, causing the reduction of infective viral particle production.	[36]
2014	Human cytomegalovirus (HCMV or HHV5)	Determination of curcumin antiviral activity against cytomegalovirus infection	Curcumin	12/5 μM, 25 μM, 50 Μm	10⁴, 10³	ND	TCID50, PCR	Decreases the serum levels of AST ^24^, ALT ^25^, CK ^26^, and LDH ^27^ in the model mice, and liver protection in HCMV-infected mice.	[37]
2014	Dengue virus (DENV)	Antiviral effects of curcumin on dengue virus type 2-infected cells	Bis-desmethoxycurcumin	29.5 μM	11.51 μM	2.56	MTT, Western blot	Decreases viral particles by suppression of the ubiquitin-proteasome system.	[38]
2014	Hepatitis C virus (HCV)	Evaluation of turmeric curcumin on entry of the hepatitis C virus	Demethoxycurcumin	5–25 μM	Does- dependent	Does-depended	MTT, RT-PCR, TCID50	Inhibition of viral entry into both hepatoma cell lines and cell-to-cell spread between neighboring cells. Curcumin also did not affect viral assembly/release of both genotypes.	[39]
2015	Herpes simplex virus 2 (HSV 2), Human immunodeficiency virus 1 (HIV-)	Determination of anti-inflammatory activity of curcumin on HIV-1 and HSV-2	Curcumin	5 μM, 50 μM	ND	ND	Trypanblue	Anti-inflammatory properties. Decreases HIV-1 and HSV-2 replication in chronically infected T-cells and primary GECs ^28^, respectively.	[40]
2015	Viral hemorrhagic septicemia virus (VHSV)	Antiviral effect of curcumin on VHSV	Curcumin	15–240 μM	ND	ND	TCID50, CCK-8, Real-time PCR, Western blot	Reduction of infective particle production. Curcumin inhibits entry of viral particles into cells by downregulating FN1 or upregulating F-actin. Curcumin inhibits viral replication by downregulation of HSC71.	[41]
2015	Human immunodeficiency virus 1 (HIV-1)	Determination of curcumin activity on HIV-1	Curcumin, Curcumin A	2 μM	35 μM, 22 μM, 0.7 μM, 0.8 μM	ND	RT-PCR, Trypanblue	Curcumin and curcumin A might affect an earlier stage of HIV-1 infection and thus indirectly reduce the subsequent HIV-1 transcription step. Both curcumin and curcumin A inhibited early LTR similarly or better than the established HIV-1 inhibitor, AZT.	[42]
2016	Human immunodeficiency virus 1 (HIV-1)	Determination of curcumin activity on HIV-1	Curcumin	20–120 μM	Dose- dependent, Time- dependent	ND	Western blot, RT-PCR	Reduction of Tat protein in infected cells, leading to inhibition of viral replication.	[18]
2016	Herpes simplex virus1 and 2 (HSV-1 and 2)	Evaluation of curcumin activity on Herpes simplex virus 1 and 2 in	Curcumin	10–100 μM	1/8 × 10 ^7^ 2/1 × 10 ^7^	ND	WST-1 assay ^29^ Plaque assay	Prevention of viral entry into vero cells. Curcumin also did not affect penetration.	[43]
2016	Human Norovirus (HuNoV)	Antiviral properties of curcumin against Norovirus	Curcumin	0/25–2 mg/mL	Dose- dependent	ND	WST-1 assay, Plaque assay, Neutralization, Real-time PCR,	Inhibition of entry or other life cycle stages rather than the replication of viral RNA.	[44]
2016	Vesicular Stomatitis virus (VSV)	Determination of curcumin effects on vesicular stomatitis virus infections	Curcumin	25–60–100 μmol	ND	ND	Plaque assay, IF	Inhibition of viral entry	[45]
2017	Hepatitis C virus (HCV)	The antiviral effects of curcumin nanomicelles on Hepatitis C virus	Curcumin, Nanomicelles	0.256 μM (highest concentration)	0.1647 mg/mL	ND	MTT, Real-time PCR	Decreases the gene expression of HCV via suppression of the Akt-SREBP-1 activation, not by NF-kB pathway. Curcumin has anti-cancer effects against anti-hepatocellular carcinoma. Inhibition of the attachment and entry of hepatitis C.	[46]
2017	Vesicular Stomatitis virus (VSV)	Determination of curcumin effects on vesicular stomatitis virus Dicer-1 Expression	Curcumin	25–60–100 μmol	ND	ND	MTT, Western blot	Antioxidant properties.	[47]
2017	Chikungunya virus (CHIKV), Zika virus (ZIKV)	Antiviral activity of curcumin against Zika and chikungunya virus	Curcumin, Bisdesmethoxycurcumin Demethoxycurcumin	11.6 μM, 16.0 μM, 13.2 μM	CHIKV: 3.89 μM 4.84 μM 0.89 μM ZIKV: 1.90 μM 3.61 μM 5.91 μM	ND	Real-time PCR, Plaque assay, Western blot	Disruption of the integrity of the viral membrane envelopes and reduction of the infectivity of viruses in a dose dependent manner.	[48]
2017	Hepatitis B virus (HBV)	Evaluation of curcumin on the hepatitis B virus replication	Bisdemethoxycurcumin	5–30 μM	Does-and time- dependent	ND	CCK8 ^7^, Western blot	Inhibition of viral replication via downregulation of cccDNA-bound histone acetylation.	[49]
2017	Human immunodeficiency virus 1 (HIV-1)	Immunomodulatory activities of curcumin-stabilized silver nanoparticles on HIV-1	Curcumin- nanoparticles	ND	ND	ND		Inhibition of NF-κB nuclear translocation and the downstream expression of the pro-inflammatory cytokines IL-1β, TNF-α, and IL-6.	[50]
2018	Enterovirus 71 (EV71)	Antiviral effects of curcumin on EV71	Curcumin	5–50 μM	ND	ND	Western blot Real-time PCR Plaque assay MTT	Inhibition of viral translation and increase of host cell viability. Decreases the phosphorylation of PKCδ ^30^ and suppression viral translation.	[51]
2018	Hepatitis C virus (HCV)	Antiviral activities of curcuma genus against Hepatitis C virus	Curcuma domestica Curcuma xanthorrhiza Curcuma heyneana	>100 μM >100 μM >100 μM	1.68 μM 4.93 μM 5.49 μM	>59.5 >20.3 >18.2	MTT, Docking	Inhibition of viral entry and interaction with viral proteins.	[41]
2018	Human T lymphotropic virus 1 (HTLV-1)	Determination of curcumin on the expression of c-FLIP in HTLV-1-associated myelopathy/tropical spastic paraparesis (HAM/TSP) patients	Curcumin	80 mg	ND	ND	Real-time PCR	Induction of apoptosis in HTLV-1 infected cells in patients with HAM/TSP ^31^.	[52]
2019	Dengue virus (DENV)	The effects of curcuminoids on dengue virus	Curcumin (CC1) Bisdemethoxycurcumin (CC2) Curcuminoids CC4, CC5, [46]	49.01 μM (CC1) 43.37 μM (CC2) 32.34 μM (CC3) 87.40 μM (CC4) 25.50 μM (CC5)	66.01 μM (CC1) 36.23 μM (CC2) 39.17 μM (CC3) 43.84 μM (CC4) 60.98 μM (CC5)	3.21 6.68 12.06 16.27 10.89	CCK-8 Plaque assay Real-time PCR	Inhibition of viral protease, resulting in suppression of DENV infectivity.	[53]
2019	Kaposi’s sarcoma-associated herpesvirus (KSHV or HHV8)	Antiviral activity of curcumin against KSHV replication and pathogenesis	Curcumin	23.56 μM	8.76 μM	2.69	EMSA ^8^	Inhibition of APE1, resulting in reduce of the transcription activity of AP-1 and NF-κB.	[54]
2019	Human immunodeficiency virus 1 (HIV-1)	Multifunctional mesoporous curcumin encapsulated iron phenanthroline nanocluster on HIV-1	Curcumin Curcumin-NIP ^9^ Curcumin-NCIP	1 mg 5 mg/mL 8 mg/mL	ND	ND	CCK-8, IF, Real time-PCR Flow cytometry, MTT	Inhibition of the release of numerous cytokines such as IL1β, IL8, TNFα ^32^, MCP1 ^33^ and MIP1α ^34^ in response to viral infection. Anti-inflammatory, anti-oxidative, and anti-HIV effects. Decreased the expression of HIV-p24 by 41%.	[55]
2019	Zika Virus (ZIKV)	Inhibitory effects of novel natural products against Zika virus	Curcumin	52.86 μM	13.67 μM	ND	ELISA, plaque assay	Disruption of the integrity of the viral membrane envelopes and reduce infectivity of viruses in a dose dependent manner.	[46]
2019	Dengue Virus (DENV)	Antiviral activity of curcumin encapsulated in nanoemulsion against Dengue virus serotypes	Curcumin (Nanocurcumin),	52.97 μM	ND	ND	MTT	Inhibition of A549 cell proliferation by inducing apoptosis	[56]
2020	Coronavirus (TGEV)	Antiviral effects of curcumin on transmissible gastroenteritis virus	Curcumin	78 μM	8.5 μM	9 μM	MTT TCID50 Western blot	Inhibitory effects on the adsorption of TGEV and virucidal activity.	[57]
2021	Human Parainfluenza Virus Type 3 (HPIV3)	Evaluation of Curcumin on Replication of Human Parainfluenza Virus Type 3	Curcumin	30 μM	Dose-dependent	ND	CCK-8 Plaque assay Real-time PCR Western blot	Inhibition of viral inclusion body (IB) formation, and virus replication by downregulate cellular PI4KB.	[58]
2021	Hepatitis B virus (HBV)	Evaluation of Curcumin on viral entry of Hepatitis B	Curcumin	30 μM	Dose-dependent	ND	MTT Real-time PCR ELISA IF	Interruption of viral entry and suppression of HBV re-infection	[59]
2021	Dengue Virus (DENV)	Antiviral activity of curcumin against Dengue virus serotypes	Curcumin	108 μM	Serotypes- dependent	ND	MTT Plaque assay	Inhibition of viral replication	[60]

^1^. cytotoxic concentration 50; ^2^. inhibition concentration 50; ^3^. selectivity index: CC50/IC50; ^4^. non-defined; ^5^. high performance liquid chromatography; ^6^. sodium dodecyl sulfate–polyacrylamide gel electrophoresis; ^7^. activator protein 1; ^8^. mitogen-activated protein kinases; ^9^. Jun activation domain-binding protein-1; ^10^. polymerase chain reaction; ^11^. 3-(4, 5-dimethylthiazolyl-2)-2, 5-diphenyltetrazolium bromide; ^12^. reverse transcription-polymerase chain reaction; ^13^. microscale therphoresis; ^14^. peroxisome proliferator-activated receptor gamma coactivator 1-alpha; ^15^. hemagglutination inhibition assay; ^16^. tissue culture infectious dose; ^17^. phosphatidylinositol 3-kinases; ^18^. nuclear factor-kappaB; ^19^. enzyme-linked immunosorbent assay; ^20^. golgi brefeldin: a resistant guanine nucleotide exchange factor-1; ^21^. phosphatidylinositol 4-kinase III beta; ^22^. ubiquitin-proteasome system; ^23^. immunofluorescence; ^24^. aspartate aminotransferase; ^25^. alanine aminotransferase; ^26^. creatine kinase; ^27^. lactate dehydrogenase; ^28^. genital epithelial cells; ^29^. water-soluble tetrazolium salt assay; ^30^. protein kinase C delta; ^31^. HTLV-1-associated myelopathy/tropical spastic paraparesis; ^32^. tumor necrosis factor-alpha; ^33^. monocyte chemoattractant protein-1; ^34^. macrophage inflammatory protein-1α.
